# Relationship Between the Ability to Detect Frequency Changes or Temporal Gaps and Speech Perception Performance in Post-lingual Cochlear Implant Users

**DOI:** 10.3389/fnins.2022.904724

**Published:** 2022-06-08

**Authors:** Dianzhao Xie, Jianfen Luo, Xiuhua Chao, Jinming Li, Xianqi Liu, Zhaomin Fan, Haibo Wang, Lei Xu

**Affiliations:** Department of Otolaryngology-Head and Neck Surgery, Shandong Provincial ENT Hospital, Cheeloo College of Medicine, Shandong University, Jinan, China

**Keywords:** cochlear implant, frequency change detection, temporal gap detection, speech perception, psychophysical test, acoustic change complex

## Abstract

Previous studies, using modulation stimuli, on the relative effects of frequency resolution and time resolution on CI users’ speech perception failed to reach a consistent conclusion. In this study, frequency change detection and temporal gap detection were used to investigate the frequency resolution and time resolution of CI users, respectively. Psychophysical and neurophysiological methods were used to simultaneously investigate the effects of frequency and time resolution on speech perception in post-lingual cochlear implant (CI) users. We investigated the effects of psychophysical results [frequency change detection threshold (FCDT), gap detection threshold (GDT)], and acoustic change complex (ACC) responses (evoked threshold, latency, or amplitude of ACC induced by frequency change or temporal gap) on speech perception [recognition rate of monosyllabic words, disyllabic words, sentences in quiet, and sentence recognition threshold (SRT) in noise]. Thirty-one adult post-lingual CI users of Mandarin Chinese were enrolled in the study. The stimuli used to induce ACCs to frequency changes were 800-ms pure tones (fundamental frequency was 1,000 Hz); the frequency change occurred at the midpoint of the tones, with six percentages of frequency changes (0, 2, 5, 10, 20, and 50%). Temporal silences with different durations (0, 5, 10, 20, 50, and 100 ms) were inserted in the middle of the 800-ms white noise to induce ACCs evoked by temporal gaps. The FCDT and GDT were obtained by two 2-alternative forced-choice procedures. The results showed no significant correlation between the CI hearing threshold and speech perception in the study participants. In the multiple regression analysis of the influence of simultaneous psychophysical measures and ACC responses on speech perception, GDT significantly predicted every speech perception index, and the ACC amplitude evoked by the temporal gap significantly predicted the recognition of disyllabic words in quiet and SRT in noise. We conclude that when the ability to detect frequency changes and the temporal gap is considered simultaneously, the ability to detect frequency changes may have no significant effect on speech perception, but the ability to detect temporal gaps could significantly predict speech perception.

## Introduction

For patients with severe-to-profound hearing loss, cochlear implantation (CI) is the most effective method for reconstructing hearing. While overall speech signal understanding has improved, there remains variability in performance across recipients, and speech perception in noise remains challenging (Firszt et al., 2004; Wilson and Dorman, 2008; Holden et al., 2013).

Understanding daily conversation depends on the ability of the auditory system to detect ongoing changes in the spectral and temporal patterns of the incoming signals (He et al., 2012). Unlike individuals with normal hearing who have approximately 3,500 inner hair cells and 12,000 outer hair cells to provide fine-grained spectral resolution, CI users rely on sound information conveyed by electrical stimulation through up to 22 electrodes (Liang et al., 2018). The real number of spectral channels used by most CI users is likely to be less than eight because of factors such as channel interactions and frequency-to-electrode mismatches (Fu et al., 2004). In addition, owing to signal processing (e.g., signal compression, bandpass filtering, and temporal envelope extraction), CI greatly attenuates the time-frequency information of sound. Furthermore, neural degeneration related to long-term deafness in CI users exacerbates their compromised ability to detect frequency differences in sound (Sek and Moore, 1995; Moore, 1996).

Exploring the influencing factors of CI users’ speech perception has always been the interest of researchers. In terms of acoustical frequency resolution or time resolution of CI users, significant correlations were reported between spectral modulation sensitivity and speech perception outcomes for CI users (Henry et al., 2005; Won et al., 2007; Anderson et al., 2012), and there were significant correlations between speech perception performance and temporal modulation detection performance measured either through sound processor (Won et al., 2011; Gnansia et al., 2014) or direct stimulation in CI users (Cazals et al., 1994; Fu, 2002). Previous study have confirmed that the ability to detect spectrotemporal modulation, covaried in both the temporal and spectral domains, was related to CI users’ speech recognition performance (Lawler et al., 2017). On this basis, some researchers (Won et al., 2015; Zhou N. et al., 2020) evaluated the correlation of speech recognition with the spectrotemporal modulation (STM) thresholds while controlling for either temporal or spectral modulation sensitivity, but different conclusions were drawn. Won et al. (2015) suggested that that slow spectral modulation rather than slow temporal modulation may be important for determining speech perception capabilities for CI users. However, Zhou N. et al. (2020) suggested that temporal information processing may limit performance more than spectral information processing in CI users. Considering the fact that similar method was applied but reached different conclusions, this study intended to use different methods to simultaneously investigate the frequency resolution and time resolution of CI users, and analyze their relative roles in predicting speech perception.

There are different approaches to measuring frequency discrimination. One of the approaches is frequency change detection, used here, examining detection of minimal frequency change within stimuli that have embedded frequency changes. The advantage of this approach is that it allows for the examination of neural response evoked by the frequency change (e.g., acoustic change complex, ACC) within stimuli (Zhang et al., 2019). Measurement of gap detection thresholds (GDTs), used here, is one of the most widely used methods for assessing temporal resolution in humans (Garadat and Pfingst, 2011; Lister et al., 2011).

Some studies have investigated the relationships between speech perception and frequency change detection or temporal gap detection. In terms of frequency change detection, a study on adult CI patients confirmed that the frequency change detection threshold (FCDT) is related to speech perception ability (Zhang et al., 2019). According to the FCDT results, the adult CI was divided into two groups: good and poor. The speech test results of the good CI group were significantly better than those of the poor CI group (McGuire et al., 2021). In a study of changing stimulated electrodes, there was a robust correlation between electrode-discrimination capacities and speech-perception performance in CI children with auditory neuropathy spectrum disorder (ANSD) (He et al., 2014). In terms of temporal gap detection, one study in young people with normal hearing and old people with hearing loss showed that, after excluding the influence of age and hearing loss, the GDT contributed to variance in speech recognition in noise (Hoover et al., 2015). However, some studies have failed to confirm the correlation between GDT and speech perception ability of CI users (Mussoi and Brown, 2019; Cesur and Derinsu, 2020). Luo et al. (2020) drew different conclusions for various subjects. For older acoustic-hearing listeners, gap detection ability was significantly correlated with SRT in noise, but this correlation was not observed in older CI users and younger listeners with normal hearing. Therefore, the relationship between gap-detection ability and speech perception requires further study.

The above FCDT or GDT are obtained by psychophysical tests. However, clinically, some CI users would not be able to participate in complicated auditory tests; therefore, it is necessary to find simple test methods to quickly estimate or predict the effects of CI. Cortical auditory evoked potentials (CAEPs), which can be recorded in a passive listening condition that does not require the participant’s attention or voluntary responses, can thus serve as a suitable tool for difficult-to-test participants (Sharma and Dorman, 2006; Small and Werker, 2012). The auditory event–related potentials (ERPs), including the onset response and the acoustic change complex (ACC), are cortically generated potentials that can be recorded from surface electrodes placed on the scalp. The onset response is typically evoked by a brief stimulus, and its presence indicates sound detection. The ACC is elicited by stimulus change(s) that occur within an ongoing, long-duration stimulation. The ACC provides evidence of discrimination capacity across various stimulus dimensions at the level of the auditory cortex (Martin et al., 2008). The ACC differs from and has advantages over the mismatch negativity (MMN), another type of auditory evoked response reflecting auditory discrimination. First, in the stimulus paradigm for the ACC, every trial of the stimuli contributes to the ACC response. In MMN recordings, a large number of standard stimuli is required to embed a sufficient number of deviant stimuli. Thus, the recording time for the ACC is much shorter than that for the MMN. Second, the ACC has a much larger amplitude (higher signal-to-noise ratio) compared to the MMN, which enables the accurate identification of ACC peaks for latency and amplitude calculation (Martin and Boothroyd, 1999). Third, the MMN is an outcome of waveform subtraction between the response to the deviant stimuli and the response to the standard stimuli, while the ACC is a response directly collected from the participant (Liang et al., 2018).

There were also some studies on the relationship between ACC induced by frequency change or temporal gap and speech perception. On ACC induced by frequency change, the N1 latency of the ACC induced by the 160-Hz tone containing a 50% frequency change was significantly correlated with the clinically collected phonetic perception score [consonant-nucleus-consonant (CNC) monosyllabic word], although a correlation between N1 latency and AzBio sentences could not be established (Liang et al., 2018). In a study of changing stimulated electrodes, compared with those with poor speech performance, the electrically evoked auditory change complex (eACC) amplitude of those with better speech performance was larger (He et al., 2014). On ACC induced by temporal gap, studies of CI or non-CI children with ANSD showed that the eACC or ACC induced by temporal gap was significantly correlated with the phonetically balanced kindergarten (PBK) word score (He et al., 2013, 2015). Unlike the above studies, a study on people with normal hearing and hearing loss showed that, after considering the influence of hearing loss, there was no significant correlation between the ACC threshold induced by frequency change and speech reception thresholds (SRTs) in noise (Vonck et al., 2021). Most of the aforementioned studies that reported a relationship between frequency change detection and speech perception did not consider the influence of hearing threshold. Therefore, the relationship between subjective or objective frequency change detection and speech recognition requires further study after excluding the influence of hearing threshold.

In addition to failing to reach a consistent conclusion, most of the aforementioned studies did not investigate frequency change detection and temporal gap detection simultaneously; therefore, it is impossible to analyze their relative effects on speech perception. Furthermore, few studies have simultaneously used psychophysical and neurophysiological methods to investigate the effects of frequency change detection and temporal gap detection on CI users’ speech perception. These were exactly what this research wanted to do.

For tonal languages, such as Mandarin Chinese, lexical tones make an essential contribution to understanding the meaning of words and sentences. Mandarin Chinese includes four tones: the high-level tone (tone 1), the rising tone (tone 2), the falling-rising tone (tone 3), and the high-falling tone (tone 4). These tones play an important role in understanding the meaning of monosyllabic words in Chinese language (Zhou Q. et al., 2020). Some studies have examined the role of temporal and spectral cues in mandarin tone recognition (Kong and Zeng, 2006; Wei et al., 2007), but few studies have examined the influence of frequency resolution and time resolution on CI users’ speech perception in Mandarin Chinese.

This study addresses the following questions: (1) whether the speech perception ability of post-lingual CI users is affected by their CI hearing threshold; (2) whether FCDT or GDT obtained using psychophysical tests could predict speech perception in post-lingual CI users of Mandarin Chinese; (3) whether ACC response induced by frequency change or temporal gap can predict speech perception in these CI users; and (4) when considering both psychophysical and neurophysiological results of frequency change detection or temporal gap detection, which factors could best predict speech perception in these CI users.

## Materials and Methods

### Participants

There were 31 CI users (11 females and 20 males; 16.3–51.4 years old; 27 unilateral and four bilateral CI users) participated in this study. Only the more satisfied side was tested in bilateral CI users, whereas the other was picked off. Bimodal CI users, who wore a hearing aid in the non-implanted ear, took off their hearing aid and had an earplug inserted into their non-implanted ear. All participants were post-lingually deafened, with a speech intelligibility rating (SIR) score above 4 (connected speech is intelligible to a listener who has little experience of a deaf person’s speech; the listener does not need to concentrate unduly). Considering that the purpose of this study was to find the relationship between CI users’ speech perception with auditory discrimination factors, and the speech perception of prelingually deafed adult CI users was greatly constrained, the participant was limited to post-lingually deafed CI users. All patients used CI for at least 6 months. All participants were native Mandarin Chinese speakers with no history of neurological or psychological disorders. Demographic data of the participants are presented in Table 1. Twenty-one participants used Chinese CIs.

**TABLE 1:** Cochlear implant (CI) users’ demographics.

CI user	Gender	Type of CI user	Age	Ear tested	Device	Duration of severe-to-profound deafness (yr)	Age at implantation	Duration of CI use (m)
01	M	Unilateral	51.88	R	Nurotron/CS-10A*	4	51.09	9.4
02	M	Unilateral	38.89	R	Nurotron/CS-10A*	10	38.21	8.9
03	M	Unilateral	39.93	R	Nurotron/CS-10A*	3	39.44	6.02
04	F	Unilateral	34.02	L	Nurotron/CS-10A*	10	33.26	9.17
05	F	Unilateral	46.42	R	Nurotron/CS-10A*	8	45.86	6.87
06	M	Unilateral	43.24	L	Nurotron/CS-10A*	3	42.49	9.79
07	M	Unilateral	18.99	L	Nurotron/CS-10A*	10	18.4	7.79
08	F	Unilateral	25.05	L	Nurotron/CS-10A*	10	24.36	9.13
09	M	Unilateral	35.82	R	Nurotron/CS-10A*	24	35.36	6.27
10	M	Unilateral	42.37	L	Nurotron/CS-10A*	5	41.83	7.43
11	M	Unilateral	47.98	L	Listent/LCI-20PI*	1	47.47	6.23
12	M	Bilateral	29.25	L	Med El/Sonata	22	28.72	6.37
13	M	Unilateral	35.16	L	Nurotron/CS-10A*	7	34.67	6.01
14	F	Unilateral	29.29	R	Nurotron/CS-10A*	2	28.75	6.41
15	F	Unilateral	29.43	R	Nurotron/CS-10A*	1	28.93	6.02
16	M	Unilateral	36.09	L	Nurotron/CS-10A*	6	35.59	6.05
17	M	Bilateral	34.79	R	Nurotron/CS-10A*	1	34.29	6.01
18	F	Unilateral	24.02	L	Nurotron/CS-10A*	13	23.49	6.34
19	M	Bilateral	37.14	L	Nurotron/CS-10A*	26	36.62	6.28
20	F	Unilateral	50.35	R	Nurotron/CS-10A*	4	49.79	6.8
21	M	Unilateral	29.78	R	Nurotron/CS-10A*	9	29.24	6.51
22	F	Unilateral	18.62	R	Med El/Sonata	11	17.69	11.24
23	M	Bilateral	19.13	L	Med El/Sonata	1	9.27	118.31
24	M	Unilateral	40.33	L	Nucleus/CI522	2	37.32	36.14
25	F	Unilateral	17.39	L	Nucleus/CI422	13	15.3	25.07
26	M	Unilateral	20.68	L	Nucleus/CI24RE(CA)	1	19.88	9.56
27	M	Unilateral	23.85	R	Listent/LCI-20PI*	1	23.04	9.69
28	F	Unilateral	25.92	L	Nucleus/CI24RE(CA)	1	24.88	12.42
29	F	Unilateral	16.25	L	Med El/Sonata	13	15.43	9.86
30	M	Unilateral	25.9	L	Nucleus/CI24RE(CA)	1	24.88	12.19
31	M	Unilateral	45.6	R	Med El/Sonata	29	45.08	6.18

*Nurotron and Listent were two Chinese domestic cochlear implant brands.

This study was approved by the Medical Ethics Committee of Shandong Provincial ENT Hospital, Shandong, China. All participants provided written informed consent before participating in the study.

### Stimuli

Stimuli in psychophysical tests and CAEPs tests were generated using Audacity software at a sample rate of 44.1 kHz and presented by the E-Prime program (Psychology Software Tools, Pittsburgh, PA, United States).

A series of tones of 800 ms duration (including 10-ms raised-cosine onset and offset ramps) at f_*base*_ of 1,000 kHz that contained different magnitudes of upward F-changes at 400 ms after the tone onset were used in frequency discrimination tests. The F-change occurred at 0 phase (zero crossing); there was no audible transient when the F-change occurred (Dimitrijevic et al., 2008; Pratt et al., 2009). The amplitudes of all the stimuli were normalized. Similar stimuli were used in some other studies (Liang et al., 2018, 2020; Zhang et al., 2019; McGuire et al., 2021).

Compared with pure tone or narrow-band noise, broadband noise can activate more electrodes in CI electrode array. Therefore, white noise was used in this study, so as to investigate the overall gap detection ability of CI users. White noise with different durations of silent gaps added in the middle position was used in the gap detection tests. There were 10 ms rising and falling periods when white noise appeared and ended. We used the 4-ms fall/rise surrounding the gap to reduce the spectral splatter, which is usually introduced by rapid onsets and offsets. To minimize the availability of intensity cues resulting from the 4-ms fall/rise, both the gap and no-gap stimuli contained a 4-ms fall/rise. GDT was affected by the duration of stimulation before and after the gap, and gap detection thresholds of older adults were markedly higher than those of younger adults for marker durations of less than 250 ms (Schneider and Hamstra, 1999). In this study, the influence of age should be excluded, so the duration of noise before and after the gap should be longer than 250 ms. In this study, the stimulation duration before and after gap was 400 ms, which was also applied in some other studies (He et al., 2013, 2015, 2018; Mussoi and Brown, 2019). In GDT testing, the stimulation duration was fixed at 800 ms, and the gap was in the middle position. In ACC testing, the duration of stimulation ranged from 800 to 900 ms, in which the gap occurred at 400 ms after the noise onset, and gap duration ranged from 0 to 100 ms.

Studies have shown that GDT was affected by the intensity of stimulation, and gap detection was known to improve with increases in stimulus intensity level until asymptotic performance was achieved (Florentine and Buus, 1984; Horwitz et al., 2011). For people with normal hearing, to achieve a stable and high level of GDT, the noise intensity should be above 50 dB SPL (Florentine and Buus, 1984) or above 20 dB SL (Horwitz et al., 2011). In this study, in order to ensure that the subjects’ GDT reaches their own high level, the noise intensity was set to not less than 70 dB SPL. Patients who were unable to tolerate sounds of 70 dB SPL were excluded from these tests.

### Procedures

The participants were tested for pulse tone hearing thresholds to ensure audibility of the stimuli presented through their clinical processors. Their speech performance was tested with 35 dB HL sound intensity above their CI hearing thresholds (average of 0.25, 0.5, 1, 2, 4 kHz pulse tone hearing threshold). They were seated on a comfortable chair in a sound-treated booth for the psychophysical and CAEP tests. Stimuli were presented in the sound field *via* a single loudspeaker placed at ear level, 1.5 m in front of the participant. The stimuli were presented at an intensity corresponding to loudness level 7 (most comfortable level) on a 0–10-point (inaudible to too loud) numerical scale to the tested CI ear (Liang et al., 2018). The intensity level of stimuli was determined separately in frequency change detection and gap detection, so the stimuli intensity level of frequency change detection and gap detection may be different for one subject. The same stimulus intensity was used for both the psychophysical and CAEP tests in frequency change detection or temporal gap detection, making the results of psychophysical test and CAEP test be comparable.

### Behavioral Tasks

#### Psychophysical Tests

An adaptive, 2-alternative forced-choice procedure was used to determine the FCDT and GDT. In each trial, a standard stimulus and target stimulus were included, and the participant was instructed to choose the target stimulus by pressing the corresponding button. The order of the standard and target stimuli was randomized and the interval between the stimuli in each trial was 0.5 s. A 2-down, 1-up staircase technique was used to track the 70.7% correct point on the psychometric function. Each response alteration was counted as response reversal. Each run generated 10 reversals. FCDT or GDT was calculated as the average of the last six reversals. The test was repeated thrice, and the average of the FCDTs or GDTs was recorded. The order of FCDT and GDT was random among different subjects.

The standard stimulus in the FCDT test was a 1,000 Hz tone, with no frequency change; the target stimulus was a 1,000 Hz tone containing a frequency change with a magnitude of up to 100%; the step size was 5% from 10 to 100% range, 0.5% from 0.5 to 10% range, and 0.05% from 0.05 to 0.5% range. The change of frequency began at 20%. The standard stimulus in the GDT test was white noise with no gap inserted, and the target stimulus was white noise with a gap inserted, in which the maximum gap duration was 100 ms; the step size was 5 ms from the 40 to 100 ms range, 2 ms from the 10 to 40 ms range, and 1 ms from the 1 to 10 ms range. The initial gap duration was 20 ms.

#### Speech Perception Tests

A computer-assisted Chinese speech audiometry platform was used to test speech perception (Xin et al., 2010). The recognition accuracy for monosyllabic words, disyllable words, and sentences in a quiet environment was tested. The SRT in noise (the SNR required for 50% correct word-in-sentence recognition in multi-talker, speech-babble noise) was only tested in participants whose recognition accuracy of a sentence in quiet exceeded 50%. In the monosyllabic words test, there were 25 syllables, and only when the consonants, vowels and tones were all correctly identified could the monosyllabic word be regarded as correctly identified. There were 40 disyllabic words in the disyllabic word test, and a single word was used as the scoring unit in the test. The sentence test in quiet consisted of 10 sentences with 50 key words, and the key word was used as the scoring unit in the test. In SRT test in noise, the initial SNR was set before test and the software could obtain the SNR corresponding to 50% correct recognition in noise by self-adapting SNR. In this study, the SRT in noisy was tested in 18 subjects. In this study, speech perception refers to the overall speech recognition ability, including the recognition accuracy of monosyllabic words, disyllabic words and sentences in quiet and the SRT in noise.

### Electroencephalographic Recordings and Data Processing

The stimuli used to induce the F-change CAEPs were tones at f_*base*_ of 1 kHz containing six different percentages of F-changes (0, 2, 5, 10, 20, and 50%). The stimuli used to induce the temporal gap CAEPs were white noise with six different gap durations (0, 5, 10, 20, 50, and 100 ms). The six stimulus conditions in tone or white noise were randomized to prevent order effects. The inter-stimulus interval was 1,200 ms. The order of the F-change and temporal gap CAEP tests was random among the participants.

The participants were seated comfortably in chairs and invited to watch silent films with subtitles. They did not need to pay attention to the sound stimulation presented *via* the loudspeaker but needed to stay awake and quiet. Electroencephalogram (EEG) results were collected using a Brain Vision (1.22) system (London, United Kingdom) and a Brain Amp DC amplifier. According to the International Standard 10–20 system, FPz, Fz, Cz, C3, and C4 were used as recording electrodes, the electrode placed at the opposite mastoid of the implanted side was used as the reference, the electrode placed between the eyebrows was the grounding electrode, and the electrode placed under the opposite eyes of the implanted side was used to record eye blinks. The electrode impedances were maintained at below 5 kΩ. The EEG was sampled at 5,000 Hz and filtered online between 1 and 100 Hz. The artifact rejection threshold was ± 120 μV. The EEG was epoched and baseline-corrected online using a window of 1,100 ms, including a 100-ms pre-stimulus baseline and a 1,000-ms post-stimulus time. For each subject, at least 200 artifact-free sweeps were recorded for each stimulation condition. These recordings were digitally filtered offline between 1 and 35 Hz before response identification and amplitude measurements.

Time windows delimiting the possible occurrence of ACC responses were determined based on the grand mean average of all recorded responses of F-change or temporal gap CAEPs. The windows for the ACC response extended from 450 to 650 ms for F-change CAEPs and from 450 to 750 ms for temporal gap CAEPs, relative to the stimulus onset. The presence of ACC was determined on ERPs based on the following criteria: (i) an expected ACC wave morphology (N1-P2 complex) within the expected time window, and (ii) a visual difference in the waveforms between the F-change conditions vs. the no-change condition or gap-inserted conditions vs. no gap-inserted conditions. Finally, the peak components of ACC (N1 and P2) were labeled. In the ACC response, the first peak, P1, is considerably smaller than the N1 and P2 peaks. The low signal-to-noise ratio of this peak makes it difficult to reliably determine the amplitude and latency of P1 (Vonck et al., 2021). The N1 potential was used to represent the ACC potential. As in previous studies (Tremblay et al., 2001; Kim et al., 2009), the N1-P2 peak-to-peak amplitude was used to represent the amplitude of the ACC.

### Statistical Analysis

The frequency change and temporal gap ACC thresholds were separately defined as the smallest frequency change and the shortest temporal gap in six change conditions that could be reliably used to evoke ACC responses. Among the six stimulus conditions, the latency and amplitude of ACC evoked by 50% frequency change and 100 ms temporal gap at Fz were used for statistical analysis. Statistical analyses were performed using the SPSS version 20.0 software (IBM, Armonk, NY, United States).

Linear regression was performed to determine whether speech perception could be predicted by FCDT/GDT, frequency change ACC thresholds/temporal gap ACC threshold, ACC latency, or amplitude induced by frequency change or temporal gap. In the cases of several significant correlations between speech perception and other measures, additional multiple stepwise regressions were conducted to assess their relative contributions to speech perception. A *p*-value less than 0.05 was used to determine factor entry into the regression model, while a *p*-value greater than 0.10 was used to determine factor removal from the model. The final model of the stepwise regression excluded any factors whose removal did not significantly impact the model fit while including any factors whose addition significantly improved the model fit. We checked for collinearity between independent variables [defined as tolerance < 0.1 and variance inflation factor (VIF) > 10] (Vonck et al., 2021).

## Results

### Correlation of Speech Perception to Cochlear Implant Hearing Thresholds

**FIGURE 1:**
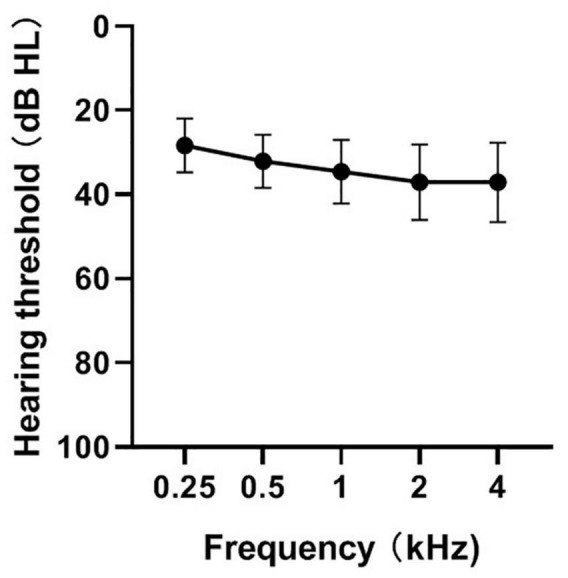
Mean hearing thresholds at frequencies of 0.25, 0.5, 1, 2, 4 kHz in CI ears (*n* = 31). The mean (circle) and the standard deviations (error bars) of the mean are plotted.

Figure 1 shows the means and standard deviations of the hearing thresholds using pulse tones at frequencies of 0.25, 0.5, 1, 2, and 4 kHz. The thresholds at the different frequencies were significantly different [*F*_(4, 120)_ = 24.09; *p* < 0.01]. A one-way repeated analysis of variance showed that the hearing threshold of 250 Hz was significantly lower than that of the remaining four frequencies (*p* < 0.01), that of 500 Hz was significantly lower than that of 1k/2k/4k (*p* < 0.01), that of 1 kHz was significantly lower than that of 2k/4k (*p* < 0.01), and that there was no significant difference between 2k and 4k (*p* > 0.05). The correlations between speech perception and hearing thresholds of 0.25, 0.5, 1, 2, and 4 kHz or the average hearing threshold of the five frequencies were analyzed. Our results suggest no significant correlation between speech perception and hearing threshold (*p* > 0.05).

### Correlation of Speech Perception to Psychophysical Measures

**FIGURE 2:**
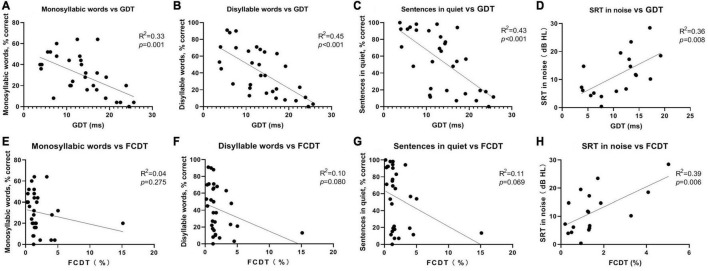
The accuracy rate of monosyllabic words **(A)**, disyllable words **(B)**, sentences in quiet **(C)** as function of GDT for all subjects. The SRT in noise **(D)** as function of GDT for partial subjects. The accuracy rate of monosyllabic words **(E)**, disyllable words **(F)**, sentences in quiet **(G)** as function of FCDT for all subjects. The SRT in noise **(H)** as function of FCDT for partial subjects.

In the simple linear regression analysis with FCDT or GDT as the independent variable and speech perception indicators as dependent variables, GDT was significantly correlated with all speech perception indicators (*p* < 0.01). FCDT was significantly correlated with SRT in noise (*p* < 0.01), but not with other speech recognition indicators (*p* > 0.05). The results are shown in Figure 2.

### Correlation of Speech Perception to Acoustic Change Complex Thresholds

In the simple linear regression analysis with the frequency change ACC threshold or the temporal gap ACC threshold as the independent variable and speech perception indicators as dependent variables, the frequency change ACC threshold was significantly correlated with the recognition rates of disyllable words (*r* = −0.41, *p* < 0.05) and sentences (*r* = −0.42, *p* < 0.05) in quiet, and with SRT in noise (*r* = 0.57, *p* < 0.05), but not with monosyllabic words (*r* = −0.27, *p* > 0.05) in quiet (Table 2A). The temporal gap ACC threshold was significantly correlated with the recognition rates of monosyllabic words (*r* = −0.43, *p* < 0.05), disyllable words (*r* = −0.44, *p* < 0.05), and sentences (*r* = −0.50, *p* < 0.01) in quiet, but not with SRT in noise (*r* = 0.09, *p* > 0.05) (Table 2B).

**TABLE 2:** Simple linear regression analysis.

	*R*	*R* ^2^	*p*
**A. Frequency change ACC threshold vs. speech performance**			
Monosyllabic words	–0.270	0.073	0.142
Disyllable words	–0.412	0.170	**0.021**
Sentences in quiet	–0.423	0.179	**0.018**
SRT in noise	0.571	0.326	**0.013**

**B. Temporal gap ACC threshold vs. speech performance**			
Monosyllabic words	–0.432	0.187	**0.015**
Disyllable words	–0.436	0.190	**0.014**
Sentences in quiet	–0.496	0.246	**0.005**
SRT in noise	0.086	0.007	0.735

**C. 50% F_change ACC potential vs. speech performance**			
Monosyllabic words	–0.008	0.00006	0.965
Disyllable words	–0.168	0.028	0.368
Sentences in quiet	–0.137	0.019	0.461
SRT in noise	–0.235	0.055	0.348

**D. 50% F_change ACC amplitude vs. speech performance**			
Monosyllabic words	0.288	0.083	0.116
Disyllable words	0.378	0.143	**0.036**
Sentences in quiet	0.385	0.148	**0.032**
SRT in noise	–0.155	0.024	0.538

**E. 100ms gap ACC potential vs. speech performance**			
Monosyllabic words	–0.343	0.118	0.074
Disyllable words	–0.400	0.160	**0.035**
Sentences in quiet	–0.403	0.162	**0.033**
SRT in noise	0.334	0.112	0.191

**F. 100 ms gap ACC amplitude vs. speech performance**			
Monosyllabic words	0.256	0.066	0.188
Disyllable words	0.445	0.198	**0.018**
Sentences in quiet	0.295	0.087	0.128
SRT in noise	–0.617	0.381	**0.008**

*The bold numbers indicate p < 0.05.*

### Correlation of Speech Perception to Potentials and Amplitudes of Acoustic Change Complex

In simple linear regression analysis with the potential or amplitude of ACC evoked by 50% F-change or 100 ms temporal gap as the independent variable and speech perception indicators as the dependent variables, the 50% F-change ACC potential had no significant correlation with any speech perception indicators (*p* > 0.05) (Table 2C); 50% F-change ACC amplitude was significantly correlated with recognition rates of disyllable words (*r* = 0.38, *p* < 0.05) and sentences (*r* = 0.39, *p* < 0.05) in quiet (Table 2D); the 100 ms gap ACC potential was significantly correlated with recognition rates of disyllable words (*r* = −0.40, *p* < 0.05) and sentences (*r* = −0.40, *p* < 0.05) in quiet (Table 2E); and 100 ms gap ACC amplitude was significantly correlated with recognition rates of disyllable words (*r* = 0.45, *p* < 0.05) in quiet and SRT in noise (*r* = −0.62, *p* < 0.01) (Table 2F).

### Multiple Regression Analyses of Speech Perception to Psychophysical Measures and Acoustic Change Complex Responses

To simultaneously consider the influence of psychophysical measures, ACC thresholds, potentials, and amplitudes of ACCs on speech perception, multiple stepwise regression analyses were conducted (see Table 3 for the results). In multiple stepwise regression, all factors related to speech perception in the simple linear regression analysis were treated as independent in the regression models.

**TABLE 3:** Multiple stepwise regression analysis.

Final mode			Included variables	β	*t*	*p*	Excluded variables	β	*t*	*p*
*R2*	*F*	*p*								
**A. Multiple regression analyses with monosyllabic words as dependent variable**										
0.331	14.336	0.001	GDT	–1.71	–3.786	0.001	Temporal gap ACC threshold	–0.212	–1.253	0.221

**B. Multiple regression analyses with disyllable words as dependent variable**										
0.686	27.331	<0.001	GDT	–3.031	–6.235	<0.001	Frequency change ACC threshold	–0.084	–0.706	0.487
							Temporal gap ACC threshold	–0.132	–1.015	0.32
			100 ms gap ACC amplitude	8.741	3.699	0.001	50% F_change ACC amplitude	0.013	0.104	0.918
							100 ms gap ACC potential	0.126	0.911	0.371

**C. Multiple regression analyses with sentences in quiet as dependent variable**										
0.55	31.841	<0.001	GDT	–3.945	–5.643	<0.001	Frequency change ACC threshold	–0.181	–1.361	0.186
							Temporal gap ACC threshold	–0.172	–1.134	0.268
							50% F_change ACC amplitude	0.153	1.147	0.262
							100 ms gap ACC potential	–0.05	–0.322	0.75

**D. Multiple regression analyses with STR in noise as dependent variable**										
0.577	9.562	0.002	100 ms gap ACC amplitude	–2.337	–3.073	0.008	FCDT	–0.068	–0.203	0.842
			GDT	0.61	2.551	0.023	Frequency change ACC threshold	0.029	0.148	0.885

In the multiple regression analysis with monosyllabic word recognition as the dependent variable, only GDT was included in the model, whereas the temporal gap ACC threshold was excluded (Table 3A). Therefore, the temporal gap ACC threshold no longer had a significant effect on monosyllabic word recognition once the effect of the GDT was considered.

In the multiple regression analysis with disyllable word recognition as the dependent variable, only GDT and 100 ms gap ACC amplitude were included in the model, whereas other factors were excluded (Table 3B). Therefore, other factors no longer had a significant effect on disyllable word recognition once the effects of the GDT and 100 ms gap ACC amplitude were considered.

In the multiple regression analysis with sentence recognition in quiet as the dependent variable, only GDT was included in the model, whereas other factors were excluded (Table 3C). Therefore, other factors no longer had a significant effect on sentence recognition in quiet once the effect of the GDT was taken into account.

In the multiple regression analysis with SRT in noise as the dependent variable, only GDT and 100 ms gap ACC amplitude were included in the model, whereas other factors were excluded (Table 3D). Therefore, other factors no longer had a significant effect on SRT in noise once the effects of the GDT and 100 ms gap ACC amplitude were taken into account.

No collinearity was found between the factors with multiple regressions for the abovementioned multiple regressions (tolerance > 0.9, VIF < 1.1).

## Discussion

### The Speech Perception of Post-lingual Cochlear Implant Users Was Not Affected by Their Cochlear Implant Hearing Thresholds

In this study, there were significant differences in CI hearing thresholds at different frequencies, and the overall trend was that the hearing thresholds gradually increased from low to high. This may be related to the individual characteristics of the study participants. Some of the study participants had long-term hearing loss and they did not wear hearing aids or the hearing aids did not adequately compensate for high-frequency sounds; therefore, they were unable to hear high-frequency sounds when they suffered from hearing loss, which made them intolerant of high-frequency sounds. Given the patient’s level of tolerance, the sensitivity to high-frequency sounds did not reach that to low frequency when mapped.

Research on people with normal hearing and hearing loss has shown that the degree of hearing loss has an important influence on speech perception (Vonck et al., 2021). In this study, correlation analyses of speech perception and different hearing frequency thresholds and mean CI hearing thresholds were conducted, but the effect of hearing thresholds on speech perception has not been confirmed. Therefore, the CI hearing threshold was excluded from the factors influencing speech perception. Nevertheless, the relationship between speech perception and CI hearing thresholds cannot be completely negated as there were no CI users with excessively poor CI hearing thresholds among the study participants. Therefore, we are only able to affirm that in the case of acceptable CI hearing thresholds, speech perception was not affected by the CI hearing threshold in post-lingual CI users.

### Speech Perception of Post-lingual Cochlear Implant Users May Not Be Affected by the Ability to Detect Frequency Change

In simple linear regression analyses, FCDT was correlated with SRT in noise, and the ACC threshold or ACC amplitude evolved by frequency change was correlated with some speech perception indicators. However, FCDT, ACC threshold, and ACC amplitude were all excluded from the subsequent multiple regression models in which, besides FCDT and frequency change ACC, GDT, and temporal gap ACC were also treated as independent variables. This indicated that FCDT or frequency change ACC did not play a significant role in explaining differences in speech perception once the effect of the GDT or temporal gap ACC was considered. This result is not in line with previous research results. Previous studies have confirmed that speech perception in CI users is related to the FCDT (Zhang et al., 2019), spectral ripple discrimination (Luo et al., 2020), and electrode discrimination ability (He et al., 2014), and that speech perception is related to the ACC response induced by frequency changes or stimulation electrode changes (He et al., 2014; Liang et al., 2018). Except for the study on spectral ripple discrimination (Luo et al., 2020), none of the aforementioned studies (He et al., 2014; Liang et al., 2018; Zhang et al., 2019) examined the temporal resolution of the subjects simultaneously. Therefore, it is uncertain whether the relationship between frequency resolution and speech perception still exists in these studies when temporal and frequency resolution were taken into account at the same time. In addition, the aforementioned studies did not rule out the possible influence of CI hearing threshold on speech perception. A study reported that the degree of hearing loss had an important influence on speech perception, and that the correlation between frequency change ACC threshold and speech perception could mainly be explained by the degree of hearing loss (Vonck et al., 2021). Therefore, the correlation between subjective or objective frequency resolution and speech perception of CI users needs to be further studied, considering both temporal resolution and CI hearing threshold.

### The Speech Perception of Post-lingual Cochlear Implant Users Was Affected by the Ability to Detect Temporal Gap

Multiple regression analysis showed that, as anticipated, speech perception could be partly predicted by the GDT and ACC amplitudes induced by the temporal gap. The auditory system uses temporal cues, such as the duration of speech segments and silent intervals between speech segments, to differentiate various speech sounds (Dorman et al., 1985). Current CIs mainly use an envelope-based speech-processing strategy to encode time-varying amplitudes in several frequency bands (Wilson et al., 1991; Shannon et al., 1995). Moreover, the spectral information provided by the CI is degraded and, therefore, significantly poorer than that heard by listeners with normal hearing (Xu et al., 2005; Sagi et al., 2009). The temporal resolution (i.e., the ability to follow rapid changes in the time waveform) is critical for speech recognition in CI users (Luo et al., 2020).

However, some studies (Mussoi and Brown, 2019; Cesur and Derinsu, 2020) have failed to confirm the correlation between GDT and speech perception. Several stimulus parameters, such as intensity, duration, temporal envelopes, and similarity of pre- and post-gate frequencies (Phillips et al., 1997; Schneider and Hamstra, 1999; Pichora-Fuller et al., 2006; Garadat and Pfingst, 2011; Horwitz et al., 2011), have also been shown to affect GDTs in CI users. The discrepancies in the literature regarding GDTs in CI users may be partially caused by the variety of stimulus parameters used in these studies (Blankenship et al., 2016). In addition to the stimulus parameters, the relationship between GDT and speech perception may also be modified by other factors affecting speech perception. Speech recognition requires cognitive processes such as attention, memory, and intelligence, as well as intact auditory pathways. The existence of various factors, such as peripheral, central, and cognitive processes, makes it difficult to evaluate the effects of temporal resolution on speech comprehension problems alone (Cesur and Derinsu, 2020). Therefore, in studies that fail to confirm the correlation between GDT and speech perception, the results may be influenced by the aforementioned stimulus parameters or factors that affect speech perception.

### Clinical Implications

This study found that GDT could significantly predict speech perception in quiet or noisy environments of post-lingual CI users. This indicated that the GDT may provide an easy, quick, and non-linguistic tool to “screen out” poor CI ears for target intervention. Interventions may include doing auditory discrimination training, designing language rehabilitation courses for specific CI users. This tool is useful when patients cannot be reliably expected to perform well on clinical speech tests (e.g., young children) or when they have language barriers (e.g., non-native speakers).

This study also found that the ACC amplitude induced by the temporal gap can significantly predict the perception of disyllabic words in quiet and speech perception in noisy. This suggests, to some extent, that ACC amplitude induced by the temporal gap can be used as an objective tool to predict speech outcomes. This tool is useful when patients cannot be reliably expected to perform well on psychophysical tests or speech tests.

### Limitations and Future Studies

Among the five conditions with frequency change or gap duration change, the minimum change condition that could induce an ACC response was defined as the frequency change ACC threshold or temporal gap ACC threshold in this study. However, this was only an approximate estimate of the actual ACC threshold. Future research can use an adaptive program and real-time data analysis to calculate the exact threshold of ACC response, as has been done by Vonck et al. (2021).

In multiple regression analysis, the ACC amplitude induced by temporal gap could significantly predict disyllabic words in quiet and SRT in noise, but not monosyllabic words or sentences in quiet. We can’t explain this result well, but it may be related to the differences among materials of speech tests. Compared with identifying monosyllabic words and sentences, identifying monosyllabic words may be a moderately difficult task. There is no hint of other syllables, so it is relatively difficult to recognize monosyllabic words. Meanwhile, it may be relatively simple to recognize the sentences in quiet because of the hints of the preceding and following words in the sentences. Therefore, the recognition of disyllabic words may better represent the recognition ability of speech sounds. But more research is needed to verify this argument.

Although GDT and ACC amplitude evoked by temporal gaps were associated with speech perception in this study, this could explain only approximately half of the variability in speech perception scores. Other factors that were not considered in the present study might help explain the remaining variability in speech perception. Cognitive abilities and listening efforts are likely to be important for speech perception. For example, according to Mussoi and Brown (2019), digit span and cognitive ability are correlated with speech perception performance.

## Conclusion

In this study, no influence of CI hearing threshold on speech perception was found in post-lingual CI users. Psychophysical and neurophysiological methods were used to investigate the influence of the ability to detect frequency changes or temporal gaps on the speech perception of post-lingual CI users. When the ability to detect frequency changes and the temporal gap was considered simultaneously, the ability to detect frequency changes had no significant effect on speech perception, but the ability to detect temporal gaps could significantly predict speech perception. The GDT obtained using the psychophysical method is a good predictor of speech perception, and the ACC amplitude induced by the temporal gap can also predict speech perception to a certain extent.

## Data Availability Statement

The raw data supporting the conclusions of this article will be made available by the authors, without undue reservation.

## Ethics Statement

The studies involving human participants were reviewed and approved by the Medical Ethics Committee of Shandong Provincial ENT Hospital. Written informed consent to participate in this study was provided by the participants.

## Author Contributions

LX, HW, and ZF contributed to the conception of the study. DX, JLu, and XC contributed to the experimental design. JLi and XL selected the data and performed the analysis. All authors contributed to manuscript revision and approved the submitted version.

## Conflict of Interest

The authors declare that the research was conducted in the absence of any commercial or financial relationships that could be construed as a potential conflict of interest.

## Publisher’s Note

All claims expressed in this article are solely those of the authors and do not necessarily represent those of their affiliated organizations, or those of the publisher, the editors and the reviewers. Any product that may be evaluated in this article, or claim that may be made by its manufacturer, is not guaranteed or endorsed by the publisher.
